# Changes of symptoms of eating disorders (ED) and their related psychological health issues during the COVID-19 pandemic: a systematic review and meta-analysis

**DOI:** 10.1186/s40337-022-00550-9

**Published:** 2022-04-13

**Authors:** Maryam Haghshomar, Parnian Shobeiri, Serge Brand, Susan L. Rossell, Ava Akhavan Malayeri, Nima Rezaei

**Affiliations:** 1 School of Medicine, Tehran University of Medical Sciences, Tehran, Iran; 2 Students’ Scientific Research Center (SSRC), Tehran University of Medical Sciences, Tehran, Iran; 3 Non-Communicable Diseases Research Center, Endocrinology and Metabolism Population Sciences Institute, Tehran University of Medical Sciences, Tehran, Iran; 4 Research Center for Immunodeficiencies, Pediatrics Center of Excellence, Children’s Medical Center, Tehran University of Medical Sciences, Dr. Gharib St, Keshavarz Blvd, Tehran, Iran; 5 Network of Immunity in Infection, Malignancy and Autoimmunity (NIIMA), Universal Scientific Education and Research Network (USERN), Tehran, Iran; 6 Center for Affective, Stress and Sleep Disorders (ZASS), Psychiatric University Hospital Basel, 4002 Basel, Switzerland; 7 Department of Sport, Exercise and Health, Division of Sport Science and Psychosocial Health, University of Basel, 4052 Basel, Switzerland; 8 Sleep Disorders Research Center, Kermanshah University of Medical Sciences, Kermanshah, Iran; 9 Substance Abuse Prevention Research Center, Kermanshah University of Medical Sciences, Kermanshah, Iran; 10 Centre for Mental Health, Faculty of Health, Arts and Design, Swinburne University of Technology, Hawthorn, VIC Australia; 11 Department of Mental Health, St Vincent’s Hospital, Melbourne, VIC Australia; 12 Faculty of Medicine, Arak University of Medical Sciences, Arak, Iran; 13 Department of Immunology, School of Medicine, Tehran University of Medical Sciences, Tehran, Iran

**Keywords:** Eating disorders, COVID-19, Confinement, Psychological consequences, Systematic review, Meta-analysis

## Abstract

**Background:**

The COVID-19 pandemic and its related social restrictions have profoundly affected people’s mental health. It can be assumed that symptomatic behaviors and mental health of individuals with eating disorders (ED) deteriorated during this time. To get a thorough overview, we conducted a systematic review and meta-analysis with the following aims: First, to provide a comprehensive overview of symptoms of ED during the COVID-19-related confinement; second, to identify psychological mechanisms which impacted the emergence and maintenance of ED symptoms; third, to describe changes of daily routine and changes of access to healthcare in individuals with ED during confinement.

**Methods:**

We searched Embase, PubMed, and Scopus databases for observational studies published between January 1st, 2020, to July 1st, 2021, which investigated the symptomatology of ED during the COVID-19 pandemic.

**Results:**

After the screening, 13 studies with 7848 participants were included in the present systematic review and meta-analysis. The overall pooled prevalence of exacerbation of binge eating, food restriction, purging behaviors, and concerns about food intake in the pooled sample of 7848 was 59.65% (95% CI: 49.30%; 69.60%), and the overall prevalence of improved symptoms of ED in the pooled sample of 741 individuals was 9.37% (95% CI: 3.92%; 16.57%). Furthermore, COVID-19-related social restrictions negatively impacted the psychological health, daily routines, and physical activity of individuals with ED. More specifically, symptoms of anxiety and depression related to ED were increased significantly over time. However, there were also positive aspects to the COVID-19 pandemic. The main positive consequences included more emotional support from the family, less pressure to engage in social activities, and more flexible meal planning. Individuals with ED reported having difficulties getting access to healthcare centers and using telemedicine. They also found a hard time communicating via online sessions.

**Conclusions:**

According to our interpretation, based on the data included in the systematic review and meta-analysis, the COVID-19 pandemic and its related social restrictions detrimentally impacted the mental health of majority of individuals with ED. Limited and impaired access to healthcare interventions appeared to have further exacerbated mental health issues of individuals with ED. Given this background, it seems that individuals with ED demand more attention during the COVID-19 crisis, and it is necessary to ensure that their course of treatment remains uninterrupted.

**Supplementary Information:**

The online version contains supplementary material available at 10.1186/s40337-022-00550-9.

## Plain English summary

The COVID-19 pandemic and the consequent lockdowns have significantly impacted people's mental health and mental status worldwide. Remarkably, people with pre-existing illnesses (e.g., eating disorders) were affected by the COVID-19-related restrictions. Thus, gathering information and data would significantly help researchers and physicians provide better future therapy and support for people with ED. Moreover, the use of online surveys to evaluate the mental status of people with ED has grown hugely in the era of the COVID-19 pandemic, which could be used as a promising way of communicating with these people in the future. Considering the growing number of studies that reported the status of individuals with ED in the COVID-19 era, we aimed to conduct a comprehensive review to summarize the current literature. Our findings show that, of all individuals participating in the surveys, 59.65% of them experienced exacerbations in their ED symptoms and 9.37% experienced improved ED symptoms. Altogether, this emphasizes the challenges to maintaining well-being in individuals with ED during the pandemic.

**Supplementary Information:**

The online version contains supplementary material available at 10.1186/s40337-022-00550-9.

## Background

The current COVID-19 pandemic has been declared a public health emergency all over the world [1]. On March 11th, 2020, the World Health Organization (WHO) recognized that the COVID-19 crisis was a pandemic. As such, the COVID-19 pandemic has affected the lives of millions of people, and its consequences on health and the economy were profound [2, 3]. Consequently, to decrease the spread of the virus and prevent further deaths and severe cases of infection, national governments, advised by health authorities, imposed confinements. To illustrate this, health authorities temporarily legislated to close borders, schools, universities, sports events, religious and cultural centers, and disallow gatherings in open spaces [4].

This public health emergency has altered conventional life at social, working, and leisure time levels. In line with this, economic concerns, lifestyle restrictions, and worries about getting affected by the COVID-19 have caused major mental health problems [3, 5–7]. As such, symptoms of anxiety, depression, and post-traumatic stress symptoms appeared to have increased during the pandemic [8]. Importantly, incidence rates of symptoms of anxiety, depression, and psychological distress raised among individuals with pre-existing mental conditions, and such an unfavorable raise was particularly observed among individuals with symptoms of eating disorders (ED) [9]. More specifically, the incidence rates of anorexia nervosa (AN), a restrictive eating disorder presented with an intense fear of weight gain and a drive for thinness, increased during the recent decades, but more so during the COVID-19-related confinement [10]. In the same vein, symptoms of binge eating disorder (BED), bulimia nervosa (BN), and mixed ED increased during the COVID-19-related confinement [11].

Eating disorders are plausibly related to physical activity patterns as a means to counterbalance the fear of gaining weight [12]. Such fears appeared to be exceptionally high among individuals with BED and AN [12]. Further, we note that confinement conditions were such that regular physical activity could have been restricted. However, results were not straightforward but mixed and most probably blurred by unassessed latent factors [13]. Despite the mixed results, plausibly, closing temporarily both outdoor and indoor sports centers should have adversely impacted people’s possibility to stay physically active. Such restrictions might have particularly negatively impacted individuals with symptoms of ED [14].

Last, COVID-19-related restrictions not only negatively impacted health anxiety and its related fear of getting affected by the COVID-19 among hospital staff [4, 15]; such conditions had an unfavorable impact also on the behavioral level to avoid postponing or canceling routine medical checks. To illustrate, among pregnant women and young mothers, higher health anxiety was associated with delaying or canceling routine health assessments [16]. To counterbalance the negative consequences of skipping on-site routine medical checks and counseling, telemedicine and online psychotherapeutic interventions were established as valuable and useful alternatives: In fact, such internet-assisted interventions showed identical results compared to traditional face-to-face psychotherapeutic interventions [17–23]. However, such online interventions need equipment, some technical abilities, and access to secure internet connections. It appears that not all people feel comfortable with such kinds of virtual and internet-based treatment and counseling. Given this, it further seems plausible that during the COVID-19-related confinement, routine treatments also decreased among people with symptoms of ED [24–26].

With this background in mind, the present meta-analysis followed these aims: First, to provide a comprehensive overview of symptoms of ED and metabolic changes during the COVID-19 confinement; second, to identify psychological challenges, changes to the daily routine; and media effects during the confinement; and third, to describe access to healthcare in individuals with ED during this special time.

We believe that the results will potentially bring insight into the psychological health condition of individuals with ED and highlight possible ways to improve their psychotherapy during the still-lasting COVID-19 pandemic its related social restrictions.

## Material and methods

We rigorously followed the algorithms of the PRISMA framework [27], and we conducted a systematic search to identify research articles on symptoms of eating disorders (ED) during the COVID-19 confinement.

### Literature search

First, we searched EMBASE, PubMed, and Scopus for research papers published up to June 2021 using the following search terms: “COVID-19” AND “Eating Disorders,” including their related keywords and medical subject headings (MeSH). Second, two independently working authors (MH and PS) screened the titles and abstracts. Third, to eliminate those papers, which did not fulfill the inclusion criteria, the full texts were thoroughly read and screened. The search strategy is presented in the Additional file 1.

### Selection criteria

We selected studies reporting at least one of the prevalent symptoms of ED, including binge eating, bulimia, and AN, and also concerns about food intake during the COVID-19 confinement. The studies contained information on subjective evaluation of AN, BN, binge eating or food restriction patterns, and concerns about food before and during the confinement in individuals with ED, compared to healthy individuals. We excluded experimental studies, and review articles.

### Quality assessments of included studies

The Newcastle–Ottawa Scale (NOS) [28] was used to assess the risk of bias. The NOS is formulated by a star allocation system, assigning a maximum of nine stars for the risk of bias in three areas: selection of study groups, comparability of groups, and ascertainment of the outcome of interest. Given that no previous validation study has been reported to provide cut-off scores, we determined that zero to three stars, four to six stars, and seven to nine stars would be considered high, moderate, and low risk of bias, respectively.

### Data extraction and management

Two independent investigators (MH and PS) performed the initial data extraction. These data included general information (authors, year), study characteristics (study groups and sample size, mean age, proportions of female participants, mean body mass index (BMI), and COVID-19 infection. In the event of incomplete data, we emailed the study's corresponding author and asked for the missing information. A third investigator checked the accuracy of the data (AAM).

### Additional analysis

Using the ‘metaprop’ function, we utilized a random-effect model for analysis to estimate Der Simonian and Laird’s pooled effect of the prevalence of patients experiencing a worsening in their ED symptoms. A forest plot was drawn to show the summary of the results of the meta-analysis and heterogeneity. Publication bias was checked by funnel plot and more objectively through Egger’s regression tests, with a *p*-value < *0.05* considered to indicate potential publication bias [29]. The trim and fill analysis was performed to evaluate the effect of publication bias by adding studies and consequently making symmetrical distribution [30]. Between-study heterogeneity was evaluated using Cochrane’s Q statistic. *I*^2^ was used to quantify between-study heterogeneity, in which a value of 0, 25, 50, and 75% represented no, low, medium, and increased heterogeneity, respectively [31]. A leave-one-out sensitivity analysis was employed to see the effect of a single study on the overall meta-analysis estimate. The result was presented in the form of text, tables, and figures. The drapery plot is a supporting figure to a forest plot and was proposed to demonstrate confidence intervals assuming a fixed significance threshold and prevent researchers from exclusively relying on the *p*-value < 0.05 significance threshold. All computations and visualizations were carried out using R version 4.0.4 (R Core Team [2020]. R: A language and environment for statistical computing. R Foundation for Statistical Computing, Vienna, Austria) and STATA 16 (StataCorp. 2019. Stata Statistical Software: Release 16. College Station, TX: StataCorp LLC) for Egger’s plots. We used the following packages: “meta” (version 4.17–0), “metafor” (version 2.4–0), “dmetar” (version 0.0–9), and “tidyverse” (version 1.3.0). All forest plots, funnel plots, and the drapery plot were designed using R. A *p*-value of < 0.05 was considered statistically significant.

## Results

### Study selection

**Fig. 1:**
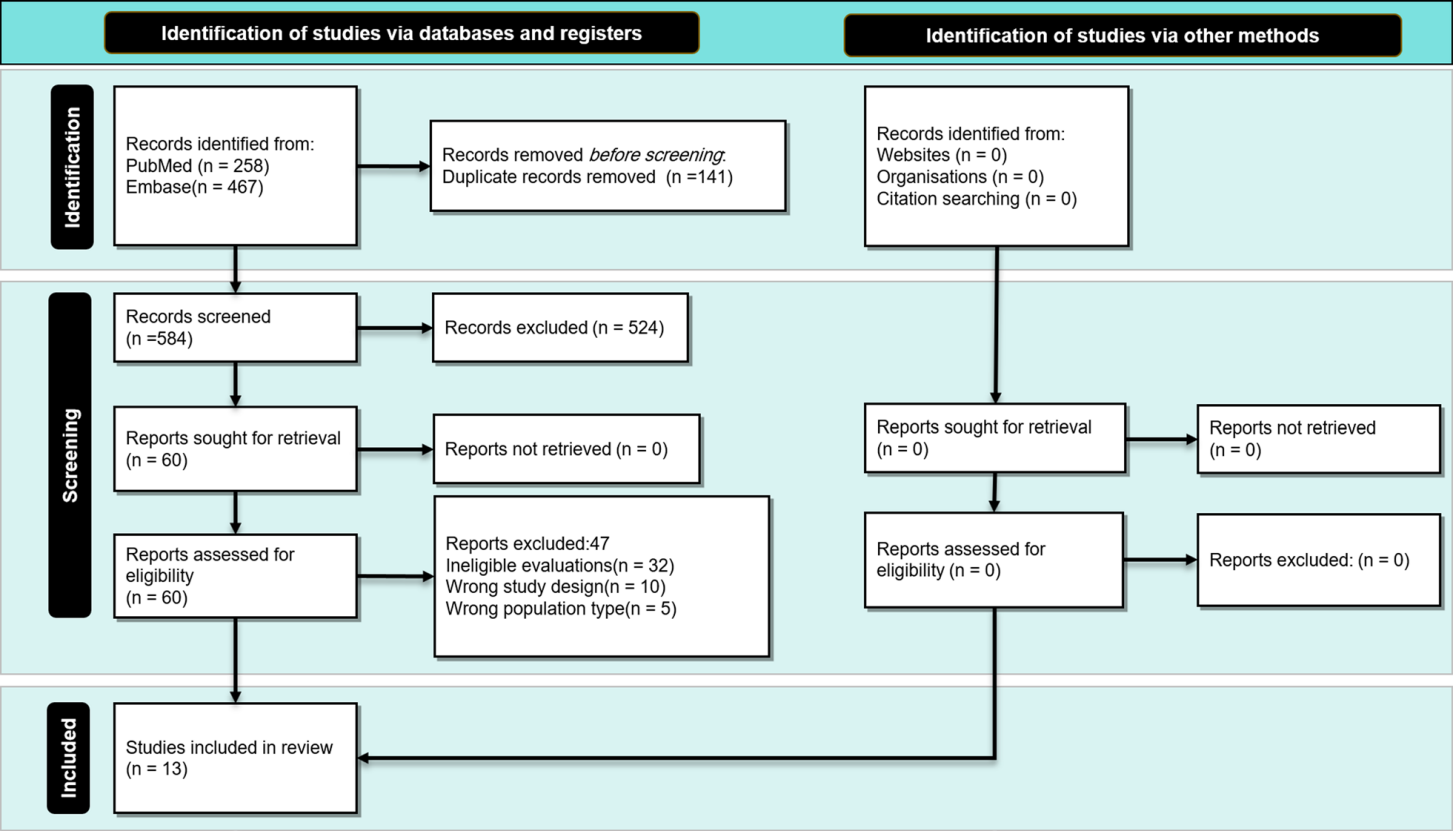
Study selection process according to the Preferred Reporting Items for Systematic Reviews and Meta-Analyses (PRISMA) guideline

After applying our strategy, we reached a total of 467 research articles. Then, we performed both the titles and abstracts screening; following this, 62 research articles were selected. Finally, we completed the full-text screening; at the end, 13 research articles were included in this systematic review and meta-analyses (Fig. 1, PRISMA diagram).

### Characteristics of included studies

Table 1 summarizes the characteristics of the 13 included studies, which were published in 2020 and 2021. Three studies included a healthy population and participants with ED [32–34]. One study included only individuals with AN [35], one only individuals with BN [36], and one only individuals with BED [37]. Other studies included in the present systematic review and meta-analysis consisted of more than one group of individuals with ED, or the type of ED was unspecified [11, 38–43]. The age of participants varied from 13–70 years. All of the included studies except one [43] were population-based, and all of them consisted of both sexes. Moreover, twelve were cross-sectional. One study was retrospective [43]. Seven studies used self-developed questionnaires [11, 33–37, 39, 41]. Two studies used the Eating Disorder Examination Questionnaire [40, 43]; two studies used the Patient Health Questionnaire [32, 42], and one study used the Rumination Response Scale for Eating Disorders [38].

**Table 1:** Characteristics of included studies

Study	Participants number	Age Mean (SD)	BMI Mean (SD)	SARSCoV-2 infection number
Schegl et al. (2020)	Adult AN (n = 112) Adolescent AN (n = 47)	25.00 (9.16) 16.26 (0.92)	17.67 (2.55) 18.22 (2.09)	N = 3 N = 0
Schegl et al. (2020)	BN (n = 55)	24.42 (6.36)	23.62 (4.58)	N = 1
Robertson et al. (2021)	Healthy (n = 125) ED (n = 35) Mental health condition (n = 104) (women = 206)	Age range: 18–79 (n = 111) Age range: 18–29 (n = 151) Age range: 30 +	NA	NA
Machitelli et al. (2020)	without a Psychiatric Diagnosis (n = 63) (women = 42) with a Psychiatric Diagnosis (n = 47) (women = 36)	47.24 (14.3) 46.38 (14.5)	40.19 (6.8) 39.88 (6.8)	NA
Phillipou et al. (2020)	ED (n = 188) (AN = 88 BN = 23) (women = 179) General population (n = 5289)	30.47 (8.19) 29.74 (7.25) 40.62 (13.67)	NA	1 10
Branley-Bell et al. (2020)	ED (n = 129) (Currently experiencing an ED = 80 Being in recovery = 49) (women = 121)	29.27 (8.99)	NA	NA
Frayn et al. (2021)	BED (n = 11) (women = 7)	42.8 (14.2)	34.7 (10.3)	NA
Vuillier et al. (2021)	AN (n = 91) (women = 7) BN (n = 46) (women = 7) BED (n = 44) (women = 7) OSFED (n = 26) (women = 7)	30.0 (9.7) 28.7 (9.1) 31.8 (9.2) 32.6 (10.7)	NA	NA
Termorshuizen et al. (2020)	**UN:** ED (n = 511) (women = 495) (AN = 318, BN = 178, BED = 156) **NL:** ED (n = 510) (women = 506) (AN = 347, BN = 117, BED = 60)	**UN:** 30.61 (9.37) **NL:** 16–60 +	NA	**UN:** 7 **NL:** 9
McCombie et al. (2020)	ED (n = 32) (women = 30) (AN = 23, BN = 3, BED = 1)	35.3 (10.3)	NA	NA
Spettigue et al. (2021)	ED-2019 (n = 43) (women = 32) COVID-triggered ED (n = 19) (women = 14) Non COVID-triggered ED (n = 29) (women = 26)	14.6 (1.79) 14.23 (1.82) 14.84 (1.76)	18.12 16.74 18.41	NA
Spigel et al. (2021)	ED (n = 73) (women = 68) (AN = 62)	19.1 (3.0)	NA	NA
Jonikas et al. (2021)	Behavioral health disorders (n = 272) (women = 155)	50 (13.5)	NA	NA

NA, not assessed; AN, Anorexia nervosa; BN, bulimia nervosa; ED, eating disorder; OSFED, Other Specified Feeding and Eating Disorders; BED, binge eating disorder; UN, United states; NL, Netherlands;

### The methodological quality of studies

The median NOS score for included studies was 5 (IQR = 2.5, mean ± SD = 6.07 ± 1.63, range: 4–9) out of 9, which shows an estimated moderate to good quality. Eight (61%) studies had high [11, 35–38, 40–42] risks of bias (scores 0–5), two (15%) had moderate [34, 39] risks of bias (scores 6–7), and three studies had (24%) [32, 33, 43] low risks of bias (scores 8–9) in their methodological quality (Table 2) [44, 45].

**Table 2:** Newcastle–Ottawa Scale (NOS) risk of bias assessment of the included studies

Author, Year	Selection (0–4)	Comparability (0–2)	Exposure/outcome (0–3)	Total score (0–9)
Branley-Bell et al. (2020)	2	0	3	5
Machitelli et al. (2020)	4	2	3	9
McCombie et al. (2020)	2	0	3	5
Phillipou et al. (2020)	4	2	3	9
Phillipou et al. (2020)	4	2	3	9
Schlegl et al. (a) (2020)	2	0	3	5
Schlegl et al. (b) (2020)	2	0	3	5
Termorshuizen et al. (2020)	2	0	3	5
Frayn et al. (2021)	1	0	3	4
Jonikas et al. (2021)	2	0	3	5
Robertson et al. (2021)	3	1	3	7
Spettigue et al. (2021)	3	2	3	8
Spigel et al. (2021)	2	0	3	5
Vuillier et al. (2021)	2	2	3	7

### Meta-analysis; Overall pooled prevalence

**Fig. 2:**
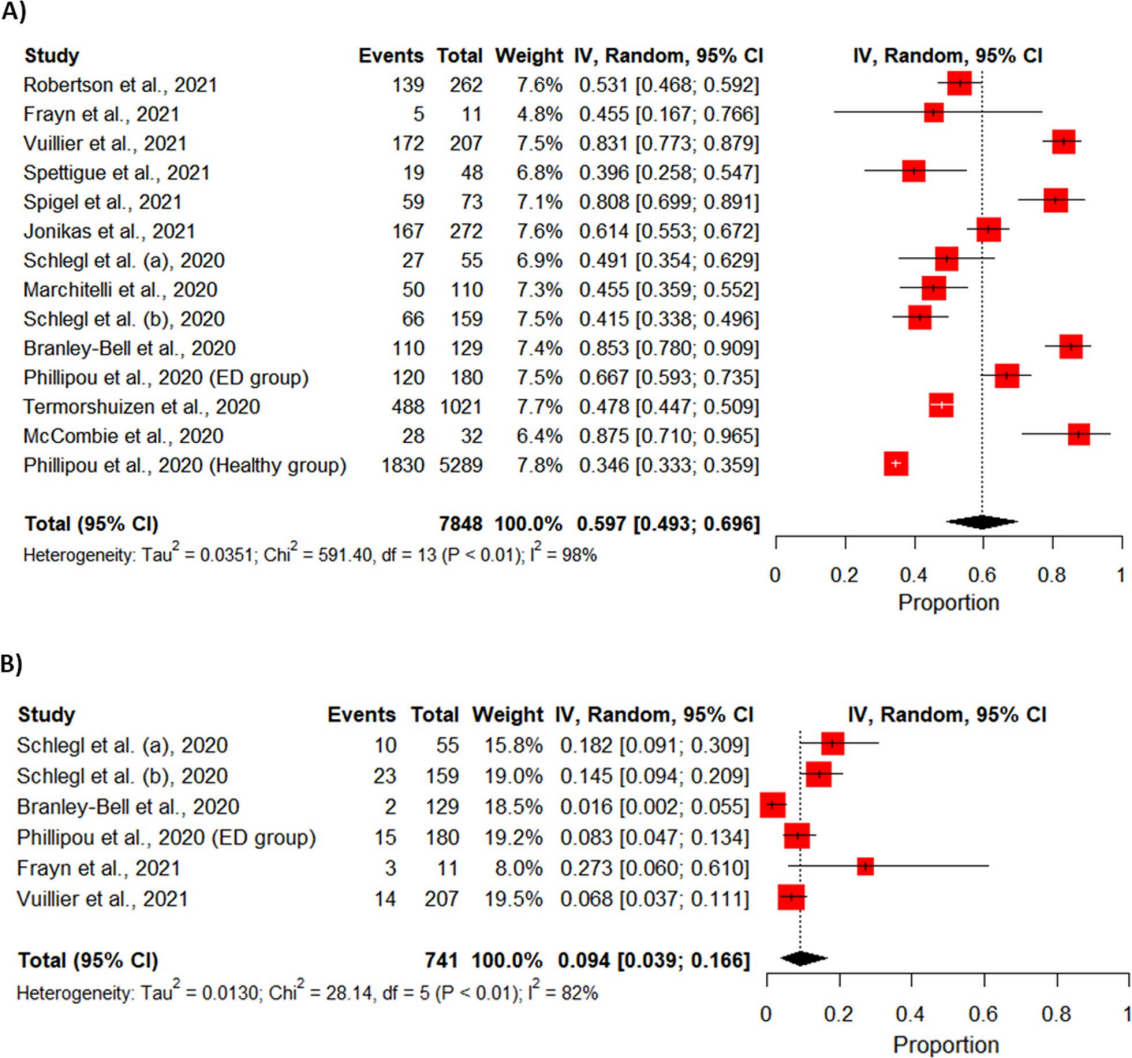
Forest plot of meta-analysis of proportions (Prevalence). **A** Deteriorated symptoms; **B** Improved symptoms

**Fig. 3:**
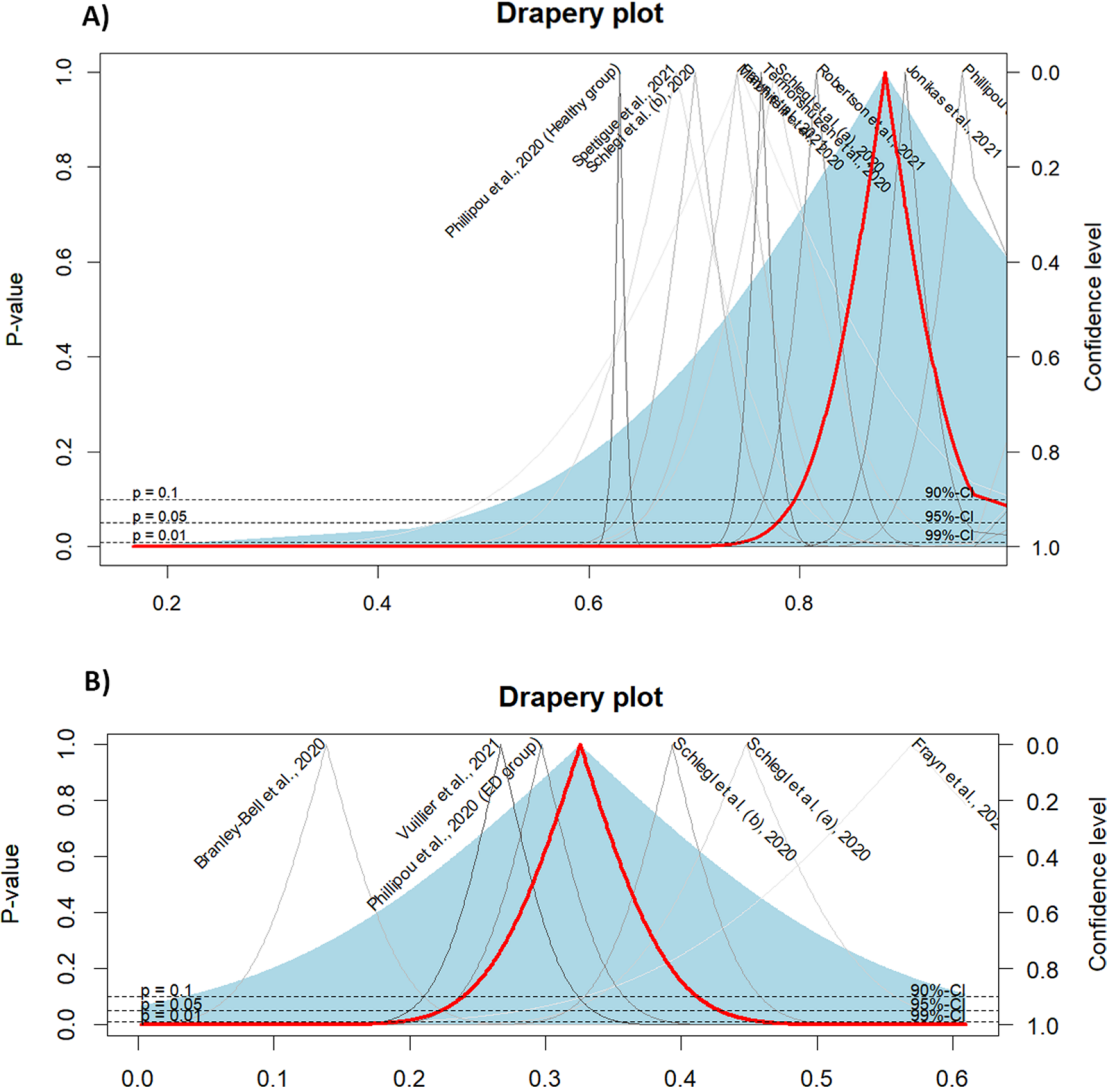
Drapery plot of meta-analysis of proportions (Prevalence). **A** Deteriorated symptoms; **B** Improved symptoms

**Fig. 4:**
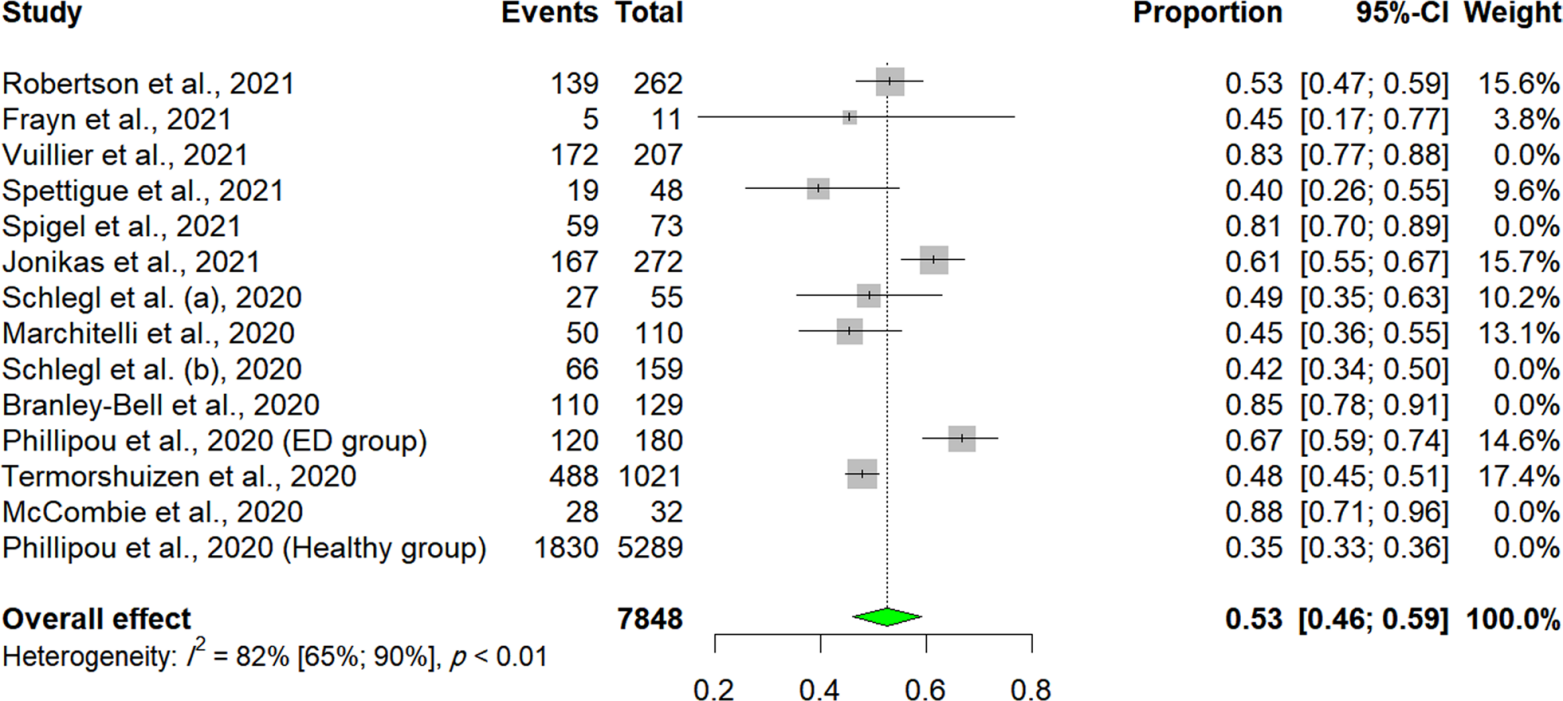
Forest plot of meta-analysis of proportions (Prevalence) removing outliers (Deteriorated symptoms)

**Fig. 5:**
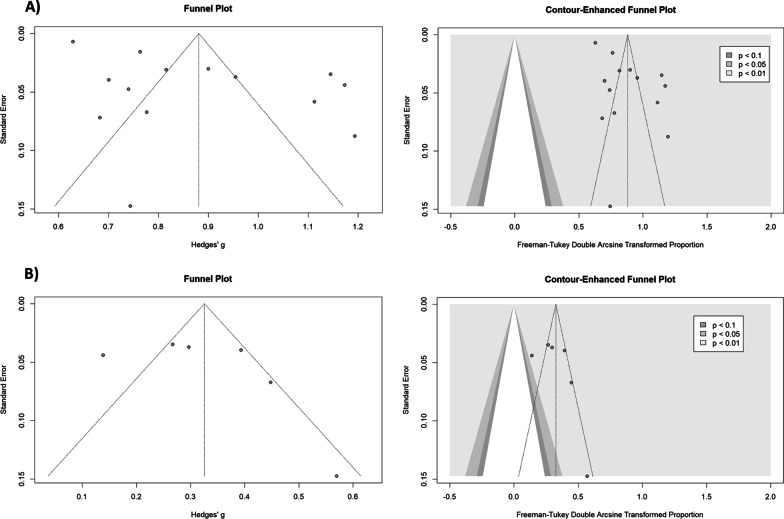
Funnel plot and counter-enhanced funnel plot of meta-analysis of proportions (Prevalence). **A** Deteriorated symptoms; **B** Improved symptoms

Cosidering the deterioration of ED Symptoms, a total of 13 studies were included in the meta-analysis. The prevalence rates of such deteriorations varied and were heterogeneous. The crude overall prevalence of deteriorated symptoms of ED in the pooled sample of 7848 individuals was 59.65% (95% CI: 49.30%; 69.60%, test of heterogeneity: *I*^2^ = 97.8%, *p*-value < 0.0001, Fig. 2A). A drapery plot is shown to visualize the meta-analysis results based on the *p*-value functions of each study (*p*-value on the y-axis and the effect size on the x-axis) (Fig. 3A). After removing the outliers [11, 32–35, 37, 42, 43], the prevalence of deterioration of symptoms of ED in the pooled sample of 1959 individuals was 52.56% (95% CI: 46.09%; 59%, test of heterogeneity: *I*^2^ = 81.7%, *p-*value < 0.0001, Fig. 4). The funnel plot (Fig. 5A) implied a publication bias for the overall crude prevalence; the Egger’s regression test confirmed the publication bias (*p* = 0.002).

Considering the improvement of symptoms, six studies were included in the meta-analysis. The overall prevalence of improved symptoms of ED in the pooled sample of 741 individuals was 9.37% (95% CI: 3.92%; 16.57%, test of heterogeneity: *I*^2^ = 82.2%, *p-*value < 0.0001, Fig. 2B). Egger’s regression test *p-*value for the meta-analysis of improved symptoms was 0.366, which does not indicate the presence of publication bias, and the funnel plot was symmetric (Fig. 5B).

### Trim and fill analysis (metatrim)

**Fig. 6:**
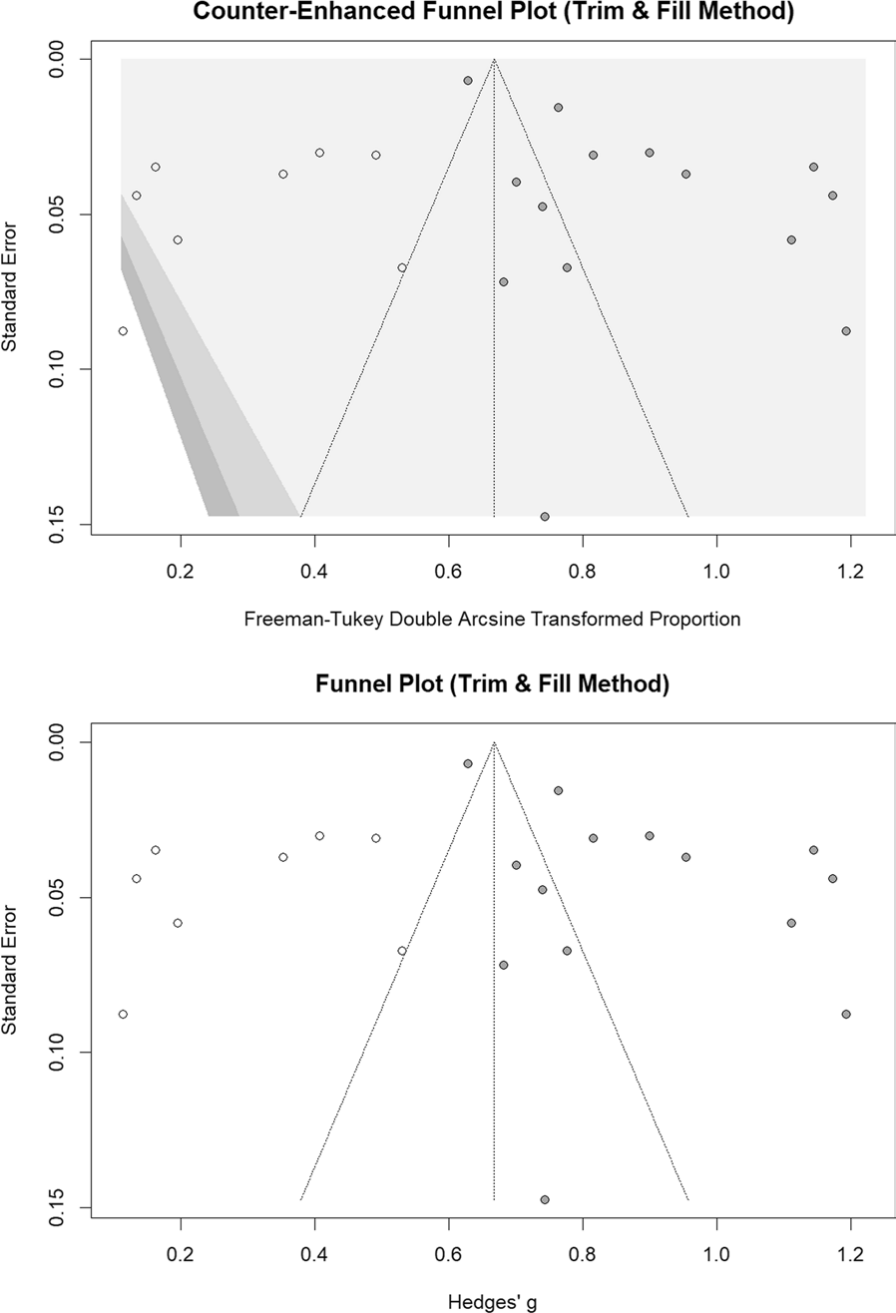
Funnel plot and counter-enhanced funnel plot of trim and fill analysis (Deteriorated symptoms)

Egger’s regression test *p*-value for the meta-analysis of deteriorated symptoms was 0.002, which confirmed the presence of a publication bias. Therefore, we performed a trim and fill analysis to investigate the impact of the publication bias, as we assumed that the funnel plot asymmetry was exclusively caused by publication bias that might not be applicable for this sort of data. The trim and fill analysis showed the presence of eight unpublished studies. Considering these studies in calculating the pooled prevalence yielded an estimated pooled prevalence of 38.24% (95% CI: 28.71%; 48.24%, test of heterogeneity: *I*^2^ = 98.3%, *p*-value < 0.0001, Fig. 6), when adjusted for the publication bias.

### Source of heterogeneity

**Fig. 7:**
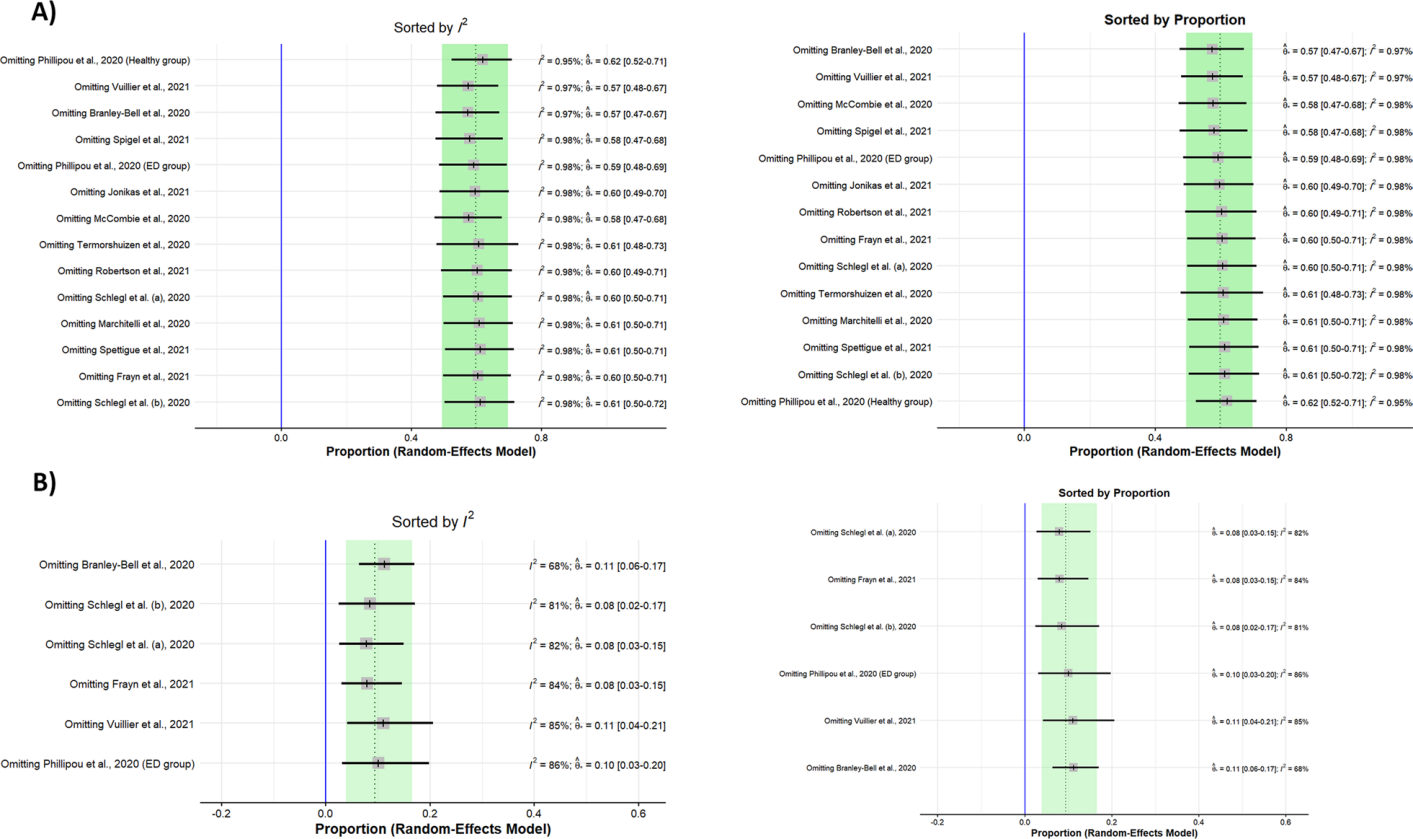
Results of Sensitivity analysis (leave-one-out analysis) of the meta-analysis (I^2^ and proportion plot). **A** Deteriorated symptoms; **B** Improved symptoms

Sensitivity analysis (leave-one-out analysis) showed that the pooled prevalence of deteriorated symptoms remained insignificant after omitting each study, and the heterogeneity did not significantly decrease (Fig. 7, Additional file 1: Fig. S1). Thus, none of the studies could explain the existing heterogeneity.

Additionally, to find the sources of heterogeneity, a meta-regression was conducted. The heterogeneity between studies could not be explained by the NOS score (*R*^2^ = 0%, *p*-value = 0.27).

**Fig. 8:**
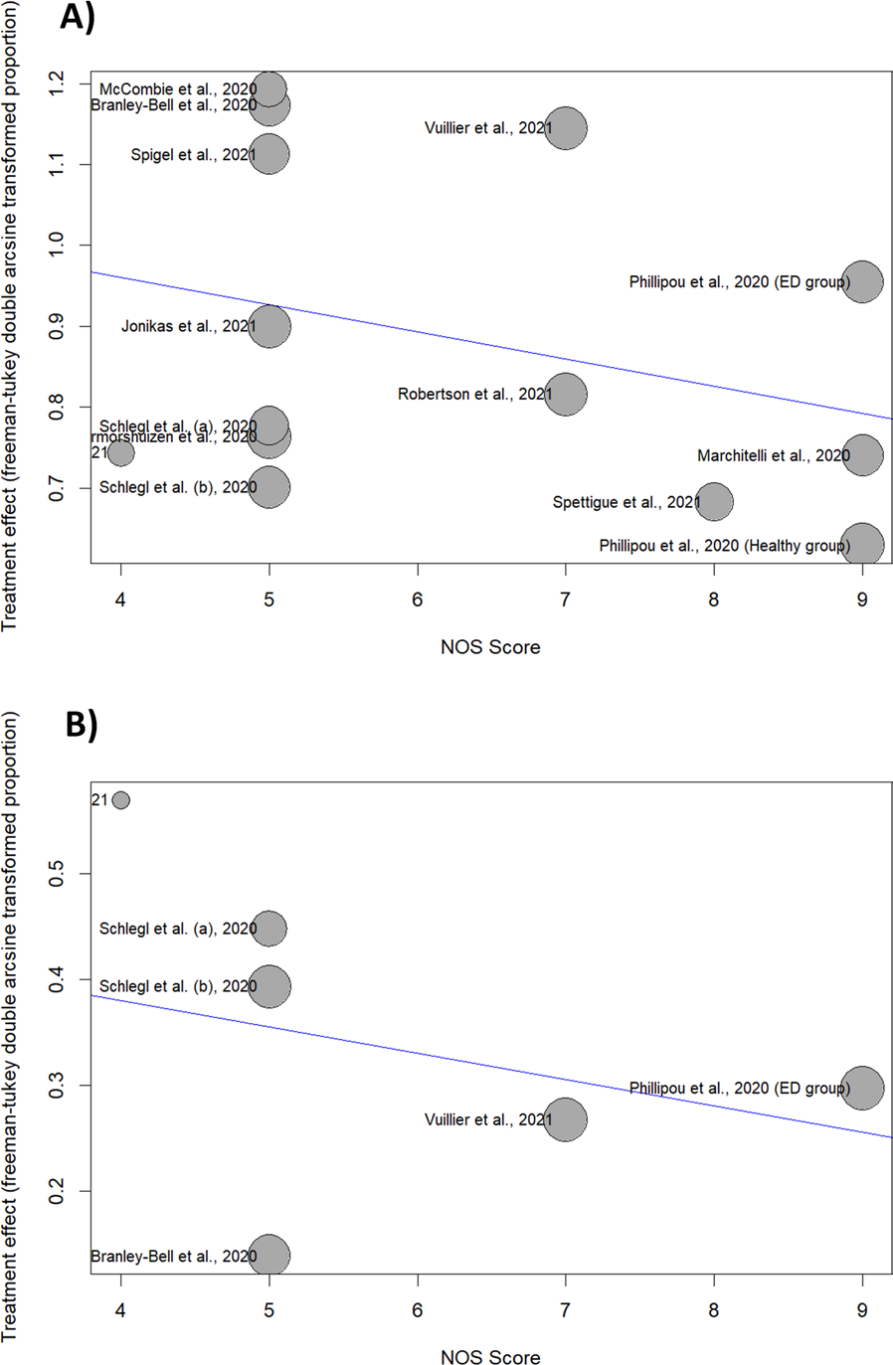
Bubble plot of meta-regression (NOS Score). **A** Deteriorated symptoms; **B** Improved symptoms

Regarding the meta-analysis of improved symptoms, sensitivity analysis showed that after omitting the study by Branley-Bell et al., the heterogeneity significantly decreased to 68%, and the pooled effect size was 11% (95% CI: 6%; 17%). Moreover, the NOS score could not explain the heterogeneity according to the meta-regression results (*R*^2^ = 0%, *p*-value = 0.48). The results of meta-regression analysis are shown as bubble plots (Fig. 8).

### Changes in patterns of binge eating

Eight studies reported increased binge eating behavior in both individuals with and without ED during the COVID-19 pandemic.

Schlegl et al. [36] used a self-developed questionnaire to assess the psychological effects of the COVID-19 pandemic among individuals with BN. More than half of these individuals reported a deterioration of their quality of life, along with a worsening of ED symptoms. More specifically, 47% of the participants experienced increased binge eating, and 36% engaged more in self-induced vomiting. Laxative use and directive abuse were also increased by almost 10%. Moreover, 40% reported the development of new BN symptoms. About 80% of the participants had more eating and weight gaining concerns. It should be noted that 18% of the participants experienced less binge eating.

Robertson et al. [34] designed an online questionnaire to explore the impact of the lockdown related to COVID-19 on eating habits, food concerns, body image, and exercise. Over 60% of the participants increased preoccupation with food intake and eating as a proxy of concerns about increased calorie intake. Furthermore, almost half of the participants were more concerned about their body image during quarantine. Women and younger people were more likely to be affected by the confinement; specifically, they were significantly more preoccupied with food, eating, and exercising. Pre-existing mental illnesses and ED were associated with different rates in “difficulty in food regulation,” “more concerned about food, eating, appearance and exercise,” and “exercising more” domains. Individuals with ED had more significant increases than participants with other mental health concerns in all of these domains except for food regulation.

Marchitelli et al. [32] investigated the psychological and psychosocial effects of the COVID-19-related confinement on obesity and weight gain among participants with and without a psychiatric diagnosis. Marchitelli et al. used a self-designed questionnaire on an online survey platform. They demonstrated that obese/overweight individuals with a psychiatric diagnosis reported statistically significantly higher depression, anxiety, stress, and binge eating scale (BES) scores than participants without a psychiatric diagnosis. Furthermore, 53% of individuals with a psychiatric diagnosis experienced increased compulsive binge eating, while nearly 40% of individuals without a psychiatric diagnosis experienced an increase in compulsive binge eating. In line with this, an increase in unhealthy food habits was also more prominent in individuals with a psychiatric diagnosis.

Phillipou et al. [33] reported that 36% of individuals with ED experienced more binge eating behaviors, and 20% reported more purging behaviors. However, 10% of participants engaged less in binge eating. Of note, also the general population had a 35% increase in compulsive binge eating.

Branley-Bell et al. [38] addressed the impact of confinement on people with recognized ED. The sample was recircuited online from Facebook and Twitter users. Around 87% of participants reported that symptoms of ED increased, while only 1.6% of the participants reported slightly better ED symptoms.

Frayn et al. [37] interviewed individuals with symptoms of BED; half of the participants reported a deterioration in their symptoms, while the other half either reported no changes or even improvements of their symptoms of BED (27%).

Vuillier et al. [39] found that 83% of individuals with ED reported an exacerbation in symptoms of ED during the COVID-19 pandemic. Precipitating factors included unfavorable changes in routine, decreased physical activity patterns, and poor emotion regulation. However, 6.8% of the participants reported fewer symptoms.

Termorshuizen et al. [11] assessed individuals with ED in the Netherlands (NL) and the United States (USA) via an online survey on social network sites. 33% of participants reported increased issues of food restriction and increased compensatory behaviors [11], compared to their behavior before the COVID-19-related confinement.

### Changes in patterns of food restriction

Five studies reported increased food restriction during the pandemic.

Schlegl et al. [35] developed a questionnaire to comprehensively investigate the psychological consequences of the COVID-19-related confinement among individuals with AN. 52% of the participants reported that their quality of life decreased, and symptoms of AN increased. Seventy-one of the cases reported increased concerns about eating and shape. In contrast, for 33% of participants, the COVID-19-related confinement did not negatively impact their symptoms. Interestingly, those participants with a decrease in symptoms of AN (14%) reported “accepting uncertainty in life” and having a “more flexible lifestyle regarding food.” Further, some participants mentioned COVID-19 as a “wake-up call.”

Phillipou et al. [33] reported that 65% of individuals with ED showed an increase in food restriction, and increasingly restrictive behavior was present in 67% of individuals with AN. Only 27% of the general population reported a greater extent of food restriction. In the meantime, 8% of the participants reported less restrictive behaviors.

Vuillier et al. [39] showed that exposure to triggering messages such as talking about weight gain/loss during confinement had a higher unfavorable impact on symptoms of ED in AN and other Specified Feeding and Eating Disorders (OSFED), compared to people with BED. In contrast, increased food availability was more harmful in BED.

Spettigue et al. [43] investigated the impact of the COVID-19-related confinement on lifestyle among youth with symptoms of ED. Nineteen out of 48 participants reported that the confinement precipitated their symptoms of ED. These participants were labeled as the “COVID-19 triggered” ED group. Compared to the “none-COVID-19 triggered ED group”, the “COVID-19 triggered ED group” reported a significantly longer symptom duration (less than 6 months vs. more than 6 months). In line with this, the “COVID-19 triggered ED group” reported significantly higher levels of eating restraint, more purging, and increased medical instability.

Spigel et al. [41] assessed adolescents with ED. 82% of participants reported increased ED thoughts and concerns, and 45% endorsed increased restrictive or compensatory behaviors [41].

### Metabolic impacts of confinement

Four studies reported changes in eating habits and BMI changes during the COVID-19 pandemic.

As a consequence of the COVID-19-related confinement, Marchitelli et al. [32] showed that 66% of obese and overweight people with a psychiatric diagnosis complained about weight gain; in contrast, weight gain was reported among 50% of obese and overweight people without a psychiatric diagnosis.

Among individuals with psychiatric diagnoses, the levels of psychological distress were not related to weight gain, while binge eating behaviors were the best predictor of weight gain. However, in those without a psychiatric diagnosis, psychological distress was significantly associated with weight gain [32].

Branley-Bell et al. [38] showed that binge eating behavior increased depending on sufficient food availability.

Spettigue et al. [43] reported that individuals with ED and sensitive to the COVID-19-related restrictions had a significantly lower BMI (16.74) compared to individuals with ED and not susceptible to the COVID-19-related restrictions (18.41). To explain this phenomenon, it appeared that the confinement increased restrictive eating behaviors and decreased the BMI among those individuals with ED and sensitive to the COVID-19-related restrictions. Further, individuals with lower BMI were at risk to report impaired mental health conditions and more restrictive eating behavior.

Jonikas et al. [42] reported that 62% of adults with behavioral health disorders also stated unfavorable changes in their eating habits.

Frayn et al. [37] found that some participants had fewer binging episodes due to reduced access to trigger foods. They mentioned that it was also easier to plan meals at home. Meanwhile, spending more time at home, they were more preoccupied with eating, which led to worsening of their symptoms.

### Psychological impacts of confinement

Eight studies found that the COVID-19-related confinement impacted both individuals negatively with and without ED, but more so on individuals with ED.

Schlegl et al. [36] reported that over 75% of individuals with BN expressed sadness, loneliness, loss of energy, sleep disturbances, and increased anxiety. Around 19% of participants reported less sadness, and 9% reported less loneliness.

Schlegl et al. [35] found that more than 70% of individuals with AN reported increased symptoms of depression and anxiety, including loneliness, sadness, the fear of not controlling their worries, worthlessness, motor and inner restlessness, and suicidal thoughts. On the other hand, around 5% of the participants manifested improved inner turmoil, less loss of control, and less concentration difficulty. In open-ended questions, participants counted more flexible meal planning and acceptance of unpredictability in life as positive aspects of the COVID-19 pandemic.

Branley-Bell et al. [38] performed a theme analysis and reported that 21% of their participants had an unfavorable change in their living situation, such as increments in their ED symptoms. Reasons were increased family pressure to eat more, difficulty hiding their ED, and more interpersonal stress. 85% of the participants experienced increased symptoms of ED related to social isolation. Disruption to routine left many participants with a feeling of lack of control and increased anxiety and symptoms of ED.

Frayn et al. [37] reported that 70% of individuals with BED stated higher general stress and anxiety, which in turn increased binge eating behavior.

Vuillier et al. [39] showed that exposure to triggering messages was the most strongly related factor to negative emotions in AN, while physical health concern was the most strongly associated factor with emotions in BN. Changes in social support and disruption to routine were accounted for BED and OSFED negative emotions, respectively. The majority of the participants reported experiencing distressing emotions, isolation, changes to routine, lack of coping skills, and loss of support that negatively impacted their ED.

Termorshuizen et al. [11] showed that 79% of the US and 66% of the NL reported being more concerned about worsening of symptoms due to the lack of structure, triggering environment, lack of social support, or lack of access to food accordant to their meal plan. In this study, participants from the US also experienced increased anxiety about exercise. These symptoms were generally consistent with the diagnosis. Moreover, 86% of the participants were concerned about their physical and mental health. Around 70% of participants suffered from a generalized anxiety disorder, and almost all reported increased anxiety due to the COVID-19 pandemic. Positive consequences of COVID-19 included increased social support and increased connection with friends and family, feeling more relaxed, and improved motivation for working.

McCombie et al. [40] found out that 88% of individuals with ED felt that their symptoms have deteriorated mainly because of the pandemic restrictions and lifestyle changes. About 66% of participants reported that their ED symptoms were worsened due to isolation which left them with more time to overthink their problems. Furthermore, it was easier to hide ED signs like weight loss in isolation. The overall rumination, depression, and anxiety rates were also increased during the confinement. However, some participants found positive aspects of lockdowns. They felt less pressure to communicate with others and had more time for self-care. Three out of four participants with autism spectrum disorder experienced a more peaceful environment and improved working situations.

Jonikas et al. [42] observed that most patients with behavioral health disorders had anxiety and depression exceeding their diagnostic threshold. These patients were more likely to experience changes in sleeping patterns, eating patterns, and living situations. Yet, some respondents found a form of resilience with minimal changes to their lives. They reported more personal time, more time with family, and an opportunity to live more authentically.

### Disruptions and restrictions to daily activities and movements

Seven studies reported altered activity and disrupted daily routine during the COVID-19 pandemic.

Schlegl et al. [36] showed that more than half of individuals with BN faced problems maintaining their daily routine. Individuals had an increased drive for activities, which mainly included walking and at-home workouts.

Schlegl et al.’s [35] study showed that more than 70% of AN patients were more concerned with physical activity, and 60% of them increased their at-home workouts and walking.

Robertson et al. [34] found that over two-thirds of the study population thought more about exercise, and half of the sample engaged more in exercising.

Phillipou et al. [33] showed that around half of the ED patients exercised more during the COVID-19 pandemic. This pattern was also present when AN patients were examined separately. 35% of the general population reported increasing daily exercise, while 43% reported less activity.

Branley-Bell et al. [38] reported a 36.5% increase in physical activity primarily driven by fear of gaining weight. However, many cases reported a reduction in their physical activity. Those who could not freely engage in purging behaviors were more likely to exercise more. Moreover, the group with a reduced physical activity engaged more in restrictive eating behaviors.

Jonikas et al. [42] found that individuals with behavioral health disorders had considerable disruptions in their daily activities and routines, including school, work and leisure areas.

Around 69% of the respondents in the McCombie et al. [40] study felt a loss of control over daily routines due to confinement restrictions which impacted their ED symptoms.

### Media effects during confinement

Five studies reported that media had deteriorating effects on ED symptoms during the pandemic.

Robertson et al. showed that women reported more changes in thoughts and behavior during the confinement [34]. An explanation for this was the increased exposure to weight stigma via social media, and these concerns were reflected in the surge of social media activity. Thousands of Instagram, Facebook, and Twitter posts contained indulgent diets or workout plans [46].

Branley-Bell et al. [38] reported that more than 80% of ED patients spent more time online, and over half of them experience worsened ED symptoms. It is interesting to note that some participants found support communities using the internet and social media.

McCombie et al. [40] found that media impact left 47% of ED patients with worse symptoms. Focus on exercising and weight loss on social media made patients more anxious about their bodies. Participants of this study were driven to compulsive exercise and unhealthy diets.

Vuillier et al. [39] explained that as the pandemic was promoted online as an opportunity to exercise more and get fit, individuals with ED went through more pressure. These messages increased the fear of gaining weight in participants diagnosed with ED.

### Healthcare utilization during confinement

Eight studies discussed the ways and the extent to which the pandemic has affected healthcare access.

Schlegl et al. [36] found that the rate of face-to-face therapy decreased from 80% before the COVID-19 pandemic to 36% during the pandemic, yet merely 20% of patients used alternative online platforms-based therapy.

Schlegl et al. [35] reported that the percentage of in-person psychotherapy dropped from 88 to 55%. Even though phone contacts and videoconference therapy rates increased, 10% of patients who used to get therapy before the pandemic did not receive any treatment.

Participants in the Branley-Bell et al. [38] study reported receiving reduced healthcare services. Some cases even felt “forgotten” by the government. All of the patients have increased ED symptoms due to the usual support networks. Many participants did not weigh themselves, which can cause unhealthy fixation on weight and directly conflict with self-monitoring required in remote treatment.

Interestingly, Frayn et al. [37] showed that most patients were more comfortable with teletherapy. An increase in flexibility in relation to the timing of sessions, reduced travel time, and easier scheduling made it a less stressful experience, and participants had more willingness to engage in online sessions. Online sessions also eased the anxiety of opening up to a therapist.

Vuillier et al. [39] found contradictory results on the efficacy of online treatment. Moreover, participants reported a feeling of detachment and a lack of connection. It was hard for some patients to find an individualized, personal, and intimate space. In contrast, online sessions demanded a lower commitment, reducing anxiety severity and reducing lengthy travel times. Vuillier et al. suggested that telehealth might be favored, as some ED patients were interested in looking at themselves in videos.

Termorshuizen et al. [11] showed that most ED patients had transitioned to online treatment, and only around 5% received face-to-face care. A large number of patients were not receiving any treatment. Face-to-face therapy was evaluated as the most excellent treatment by the patients, and more than half of the respondents reported worse quality of treatment.

Spettigue et al. [43] reported a surge in youth requiring inpatient treatment. Moreover, the number of outpatient referrals and emergency ED cases drastically increased.

Spigel et al.’s [41] study showed that access to care remained high during the pandemic. However, almost half of the patients reported stopping at least one aspect of their treatment, and around 32% of telehealth users found it worse than usual treatment. Those who had treatment disruption experienced increased destructive ED thoughts.

## Discussion

Our meta-analysis results revealed that the overall crude prevalence of deteriorated symptoms of ED in the pooled sample of 7848 individuals was 59.65%. After removing the outliers, the prevalence of deterioration of symptoms of ED in the pooled sample of 1959 individuals was 52.56%. Interestingly, the proportion of improved ED symptoms was 9.37% in the pooled sample of 741 subjects. Our findings also clarify critical themes of how the COVID-19 pandemic affected individuals’ lives struggling with ED. As such, we have primarily focused on the negative aspects of the pandemic.

Earlier reviews suggested that individuals with pre-existing mental disorders, including ED, were more vulnerable to pandemic changes and had an increased risk of symptoms like anxiety, depression, and insomnia [47, 48].

A cohort study compared a sample of 48 individuals assessed from April 1st to October 31st, 2020, with a sample of 43 youth studied during the same time frame but in 2019 to specify the impacts of the pandemic. The sample in the pandemic presented with a significantly lower treatment goal weight than the sample assessed in the previous year. They also performed worse in the clinical impairment assessment questionnaire. Overexercising occurred in 46.5% of individuals evaluated in 2019 and 73.7% in the pandemic group. While only 2.3% of 2019 participants reported purging symptoms, more than 20% of the pandemic cohort reported purging symptoms. Those in the COVID-19 cohort reported significantly higher eating restraint compared to the 2019 cohort group. Those who were assessed during the pandemic were significantly more medically unstable than those evaluated during 2019. Finally, within four weeks of assessment, the hospital admission rates were 41.9% in 2019 and 70.8% in 2020. There was a 63% increase in individuals requiring inpatient treatment during 2020 compared to 2019. There was also a surge of 28% in individuals with ED who were referred to the emergency room during the COVID-19 period compared to the previous year [43].

To better understand the natural course of ED, Grilo et al. performed a 5-year prospective study and assessed the probability of BN and OSFED remissions and relapses. By 60 months, the likelihood of remission was 74% for BN and 83% for OSFED. The possibility of relapse for those who went through remission was 47% for BN and 42% for OSFED [49]. Another study found that the probability of remission was 69% for AN and 55% for BN, and most individuals with ED suffer for 1–15 years [50]. Though the information on the one-year course and outcome of ED is limited and highly dependent on the current stage of the disease, it can be assumed that some individuals go through remission in a year, and some experience relapse. According to the 5-year course, rates of remission are higher than relapse. In the current study, we found that during the COVID-19 confinement, most individuals with ED are at higher risks of symptom deterioration, while a few experience improved symptoms.

Our findings are generally in line with these preliminary data. However, although a considerable portion of individuals reported an increase in ED symptoms, some reported no change or slight changes in their symptoms. A few even reported an improvement in their ED symptoms during the confinement than before. Moreover, most individuals were able to maintain their weight. Though highly speculative, we claim that the following reasons can explain this finding. Several individuals were former inpatients, and they might have developed coping skills during their treatment. Most of the individuals continued their psychotherapy during the pandemic, which was likely influential in preventing relapse. These individuals with AN had relatively higher BMI, which reflects their self-efficacy in ED management. A subset of patients might have experienced more social interaction with their parents and had more regular meals during the confinement. Spending time in the quarantine may have given patients better control over the food environment and ameliorated binge eating symptoms. Less pressure to engage in events and social interactions and having more personal space were also among the positive aspects of the confinement. Overall, findings suggest that the consequences of the COVID-19 crisis were highly heterogeneous.

One overall result, in line with previous studies, was that it appeared people with ED used more dysfunctional coping strategies than healthy individuals, most probably due to more impaired emotion regulation [39]. We inferred that challenges caused by the COVID-19 pandemic appeared to have increased ED symptom deterioration, and we have shown that BED occurred more frequently during the COVID-19 pandemic.

AN patients had a low level of openness to new situations and avoided uncertainty [51]. The present COVID-19-related confinement appeared to increase their anxiety and left them with a feeling of helplessness. Thus, during the confinement, AN patients were more likely to experience weight and body shape concerns than before, which may have caused increased food restriction.

Social isolation, anxieties about accessing particular food and medication, and significant changes to the daily routine may trigger disordered eating behaviors. Working from home and the closure of gyms and other sports facilities also lead to less physical activity, harming general health [33]. Spending more time at home facilitates more preoccupation with food. These factors have led to metabolic changes and weight gain during the pandemic [52].

After declaring the global pandemic and imposing social distancing rules, a significant increase in anxiety and depression was observed in the general population [53]. Moreover, financial problems, fear of contagion, and uncertainty towards the future increased drastically [54]. These issues raise concerns for the group with existing mental illnesses and the psychological impacts of the pandemic on them.

The increase in anxiety and depression appeared to interact with ED symptoms in a vicious cycle, and attempts to deal with depression and anxiety often exacerbated ED symptoms. In this regard, the following groups were more vulnerable: Unmarried women were more likely to experience depressive symptoms [55]. Low resilience to uncertainty has been recognized as a risk factor for symptoms of anxiety and depression [56].

Studies showed that mental well-being, perceived stress, social support, and control scores are significantly better in ED patients in recovery [57]. As discussed, the confinement may worsen all these measures, meaning that the COVID-19 crisis will make many ED patients vulnerable to relapse.

Social networks are changing from an amenity to a necessity. Studies showed that ED concerns were promoted by increased use of social media. Higher amounts of time spent on social platforms bore the risk of seriously distorting body image and leading to worse eating habits [58]. Social isolation appeared to be an essential trigger to spend more time online. Self-improvement messages shared online reinforced thin idealization and excessive dieting and exercising [39]. Adolescents experience more psychological impairment and medical instability. This might be due to the increased social media time, more unstructured spare time, and isolation from peers in the youth [43].

The COVID-19 pandemic may have caused a lowered access to care and limited social support. This happens when patients are more vulnerable than ever with a perceived lack of control and high health anxieties. A preliminary analysis in a group of AN patients suggested that many of them could not tolerate seeing themselves on screens. Their families expressed that online meetings lack non-verbal communication, and it was hard for patients to handle their weigh-ins [59].

Although the COVID-19 has been considered a turning point for telehealth (24), online sessions have still not compensated for in-person psychotherapy [17–20, 23, 60]. Some ED patients still have an ambivalent feeling toward online interventions [59]. Furthermore, some therapists appeared not to feel professional or professionally prepared to use new electronic technologies.

Prolonged confinement and the stresses and difficulties of living in challenging environments may be seriously harmful for patients coping with ED.

One interesting point is that Termorshuizen et al. conducted a large-scale study in the US and the Netherlands. Despite the different approaches to pandemic control and other COVID-19 positive cases, ED patients experienced similar symptoms and mental well-being. Their result is in line with the literature. However, all of the studies included here are performed in developed countries where most people have access to adequate healthcare.

## Limitations

Despite the novelty of the results, the following limitations should be considered, and we should remain cautious in interpreting these results. First, the population analyzed in the meta-analysis was heterogeneous, consisting of ED patients (AN, BN, binge eating), other mental disorders, and the healthy population. This heterogeneity might have blurred the overall pattern of results. Second, only papers published in English were considered, while publications in other languages were not considered. Third, data were recruited via online platforms in all of the studies; this results in biased samples of individuals who have access to technology. Moreover, data recircuited online potentially bias the results toward more negative results, as individuals experiencing worse symptoms tend to engage more in questionnaires. Spigel et al. found that participants with restrictive ED, generally perceived as a more severe case than binge eating, responded more to surveys [41, 61, 62].

## Future directions

Future longitudinal studies are required to determine whether increased depression or anxiety are driving ED symptoms or whether already disordered patients are using unhealthy eating behavior as a coping mechanism in a situation with a feeling of lack of control. This issue has significant implications in understanding how severe psychosocial stressors can affect ED symptomatology across the population. This understanding of mechanisms related to eating habits will guide us toward more targeted and more competent public health decisions to manage ED. Moreover, future methods to improve online treatment should be introduced.

## Conclusions

In summary, based on the findings of this study, we assumed that the COVID-19-related confinement has made a large portion of individuals with ED more vulnerable, and they are at risk of deterioration of ED symptoms and general mental problems. Alternative treatment modalities and better promotion of e-health should be considered to deal with ED symptoms and prevent ED relapse. Moreover, heterogeneous results on symptoms deterioration highlight the importance of an individualized approach in treating each patient to address relevant concerns.

## Supplementary Information

**Additional file 1: Fig. S1.** Results of Sensitivity analysis (leave-one-out analysis) of the meta-analysis (Baujat Plot & Influence Diagnostics). **A** Deteriorated symptoms; **B** Improved symptoms

## Data Availability

Not applicable.

## Abbreviations

AN: Anorexia nervosa
BED: Binge eating disorder
BN: Bulimia nervosa
ED: Eating disorder
ED: Eating disorder
OSFED: Other Specified Feeding and Eating Disorders
