# Supplementary material for: Changes of symptoms of eating disorders (ED) and their related psychological health issues during the COVID-19 pandemic: a systematic review and meta-analysis
Source: J Eat Disord. 2022 Apr 13;10:51. doi: 10.1186/s40337-022-00550-9 (PMC9006500; doi:10.1186/s40337-022-00550-9)
Supplement: Supplementary file 1 — Additional file 1: Fig. S1. Results of Sensitivity analysis (leave-one-out analysis) of the meta-analysis (Baujat Plot & Influence Diagnostics). A Deteriorated symptoms; B Improved symptoms [file 40337_2022_550_MOESM1_ESM.docx]

**Supplementary material**

**Title: Assessment of eating disorders risk and symptoms during the COVID-19 pandemic: A systematic review and meta-analysis**

**Authors:** Maryam Haghshomar, Parnian Shobeiri, Serge Brand, Susan L. Rossell, Ava Akhavan Malayeri, Nima Rezaei

***Corresponding author:**

Nima Rezaei, MD, Ph.D., Research Center for Immunodeficiencies, Children’s Medical Center, Dr. Gharib St, Keshavarz Blvd, Tehran, Iran, E-mail: [rezaei_nima@yahoo.com](mailto:rezaei_nima@yahoo.com)

**Table of Contents**

Search Strategy and Keywords2

Supplementary Figure S13

1. **Search Strateg****y and Keywords**

| #1 | (COVID 19[Title/Abstract]) OR (COVID-19 Virus Disease[Title/Abstract]) OR (COVID 19 Virus Disease[Title/Abstract]) OR (COVID-19 Virus Diseases[Title/Abstract]) OR (Disease, COVID-19 Virus[Title/Abstract]) OR (Virus Disease, COVID-19[Title/Abstract]) OR (COVID-19 Virus Infection[Title/Abstract]) OR (COVID 19 Virus Infection[Title/Abstract]) OR (COVID-19 Virus Infections[Title/Abstract]) OR (Infection, COVID-19 Virus[Title/Abstract]) OR (Virus Infection, COVID-19[Title/Abstract]) OR (2019-nCoV Infection[Title/Abstract]) OR (2019 nCoV Infection[Title/Abstract]) OR (2019-nCoV Infections[Title/Abstract]) OR (Infection, 2019-nCoV[Title/Abstract]) OR (Coronavirus Disease-19[Title/Abstract]) OR (Coronavirus Disease 19[Title/Abstract]) OR (2019 Novel Coronavirus Disease[Title/Abstract]) OR (2019 Novel Coronavirus Infection[Title/Abstract]) OR (2019-nCoV Disease[Title/Abstract]) OR (2019 nCoV Disease[Title/Abstract]) OR (2019-nCoV Diseases[Title/Abstract]) OR (Disease, 2019-nCoV[Title/Abstract]) OR (COVID19[Title/Abstract]) OR (Coronavirus Disease 2019[Title/Abstract]) OR (Disease 2019, Coronavirus[Title/Abstract]) OR (SARS Coronavirus 2 Infection[Title/Abstract]) OR (SARS-CoV-2 Infection[Title/Abstract]) OR (Infection, SARS-CoV-2[Title/Abstract]) OR (SARS CoV 2 Infection[Title/Abstract]) OR (SARS-CoV-2 Infections[Title/Abstract]) OR (COVID-19 Pandemic[Title/Abstract]) OR (COVID 19 Pandemic[Title/Abstract]) OR (COVID-19 Pandemics[Title/Abstract]) OR (Pandemic, COVID-19[Title/Abstract]) OR ("COVID-19"[Mesh]) |
| --- | --- |
| #2 | ("Feeding and Eating Disorders"[Mesh]) OR ("Feeding and Eating Disorders") OR (Feeding Disorders) OR (Disorder, Feeding) OR (Disorders, Feeding) OR (Feeding Disorder) OR (eating disorders) OR (Disorder, Eating) OR (Disorders, Eating) OR (eating disorder)) OR (Appetite Disorders) OR (Appetite Disorder) |
| #3 | **#1 AND #2**  ((((((((((((((((((((((((((((((((((((COVID 19[Title/Abstract]) OR (COVID-19 Virus Disease[Title/Abstract])) OR (COVID 19 Virus Disease[Title/Abstract])) OR (COVID-19 Virus Diseases[Title/Abstract])) OR (Disease, COVID-19 Virus[Title/Abstract])) OR (Virus Disease, COVID-19[Title/Abstract])) OR (COVID-19 Virus Infection[Title/Abstract])) OR (COVID 19 Virus Infection[Title/Abstract])) OR (COVID-19 Virus Infections[Title/Abstract])) OR (Infection, COVID-19 Virus[Title/Abstract])) OR (Virus Infection, COVID-19[Title/Abstract])) OR (2019-nCoV Infection[Title/Abstract])) OR (2019 nCoV Infection[Title/Abstract])) OR (2019-nCoV Infections[Title/Abstract])) OR (Infection, 2019-nCoV[Title/Abstract])) OR (Coronavirus Disease-19[Title/Abstract])) OR (Coronavirus Disease 19[Title/Abstract])) OR (2019 Novel Coronavirus Disease[Title/Abstract])) OR (2019 Novel Coronavirus Infection[Title/Abstract])) OR (2019-nCoV Disease[Title/Abstract])) OR (2019 nCoV Disease[Title/Abstract])) OR (2019-nCoV Diseases[Title/Abstract])) OR (Disease, 2019-nCoV[Title/Abstract])) OR (COVID19[Title/Abstract])) OR (Coronavirus Disease 2019[Title/Abstract])) OR (Disease 2019, Coronavirus[Title/Abstract])) OR (SARS Coronavirus 2 Infection[Title/Abstract])) OR (SARS-CoV-2 Infection[Title/Abstract])) OR (Infection, SARS-CoV-2[Title/Abstract])) OR (SARS CoV 2 Infection[Title/Abstract])) OR (SARS-CoV-2 Infections[Title/Abstract])) OR (COVID-19 Pandemic[Title/Abstract])) OR (COVID 19 Pandemic[Title/Abstract])) OR (COVID-19 Pandemics[Title/Abstract])) OR (Pandemic, COVID-19[Title/Abstract])) OR ("COVID-19"[Mesh])) AND (((((((((((("Feeding and Eating Disorders"[Mesh]) OR ("Feeding and Eating Disorders")) OR (Feeding Disorders)) OR (Disorder, Feeding)) OR (Disorders, Feeding)) OR (Feeding Disorder)) OR (Eating Disorders)) OR (Disorder, Eating)) OR (Disorders, Eating)) OR (Eating Disorder)) OR (Appetite Disorders)) OR (Appetite Disorder)) |

**Supplementary Figure S1. Results of Sensitivity analysis (leave-one-out analysis) of the meta-analysis (Baujat Plot & Influence Diagnostics)**

**
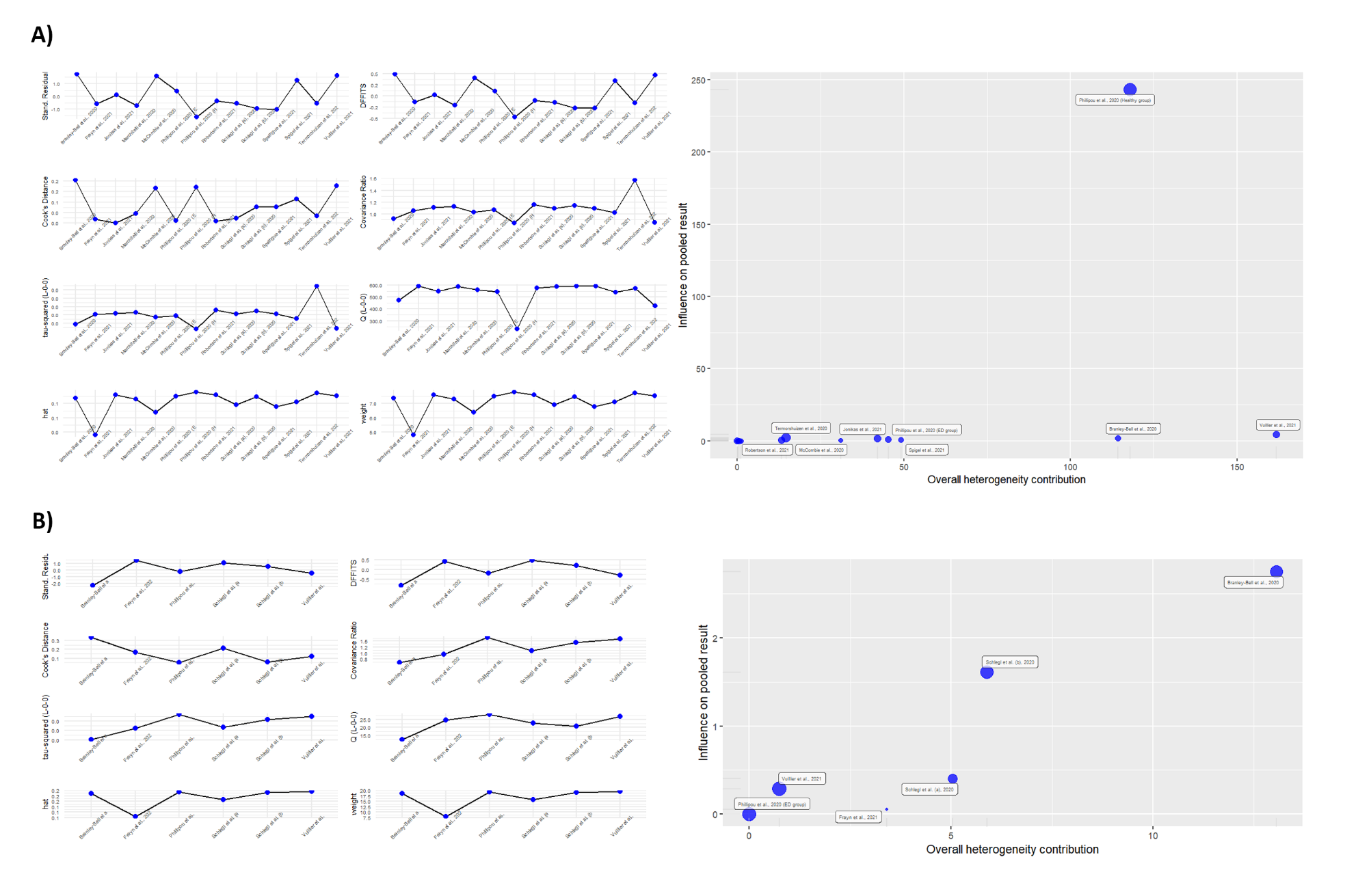
A: Deteriorated symptoms; B: Improved symptoms**
